# Hospitals and the Sunday Fund

**Published:** 1911-06-18

**Authors:** 


### HOSPITALS AND THE SUNDAY FUND.

Hospital Sunday has become an institution in this country, and, indeed, it would be an ill time for the voluntary hospitals on the one hand and the public on the other when the community ceased to take an interest in this annual expression of practical goodwill towards organised charity in aid of the sick poor. Every year the hospital problem is getting more difficult, not because, as some people allege, the voluntary system that has hitherto been of such incalculable service to the country is on its last legs; not because the people no longer care to support voluntary charities, but simply because conditions and circumstances are changing, and the various factors which add to the difficulties under which the work of the hospitals is carried on are tending to increase.

### Why Hospital Expenses have Increased.

The cost of running a modern hospital has gradually increased during the last ten years because it is imperative that a hospital must march with the times. It must not stagnate; it must make use of the latest and most reliable apparatus, not merely for purposes of treatment, but for diagnosis as well; it must provide special departments, and constantly increase its efficiency by adding to its resources and accommodation. It is only by paying attention to these points that it can keep its place on the register of modern institutions which are thoroughly efficient, and efficiency is the first and most obvious test of the usefulness of a hospital. Our modern methods of treatment demand the use, very often, of expensive apparatus and costly drugs; ten years ago the *x*-ray department was badly equipped and arranged; to-day a hospital without a proper radiographic annexe must be looked upon as one that is inadequately equipped for the work that it has to do. Similarly the eye department, the radium and actino-therapeutic department, and the bathing and massage departments demand special and costly apparatus which must be installed if the institution would claim to rank as efficient. All this costs money, for new scientific apparatus is always expensive since the demand for it is necessarily limited.

### The Increased Scope of Hospitals.

Again, the usefulness of hospitals has increased; they now deal with a much larger percentage of cases than they dealt with a decade or two ago. The up-to-date, thoroughly efficient hospital is the one which benefits the largest number of patients in the shortest possible time, but the very fact that it deals with more patients than formerly has increased the annual cost of upkeep. When a patient leaves his hospital bed, cured, and fit to resume his work, or at least relieved and able to go to a convalescent home or back to his own house, another patient is almost at once put into his place. But before that is done the bed must be aired, disinfected, cleaned; new bedding and bedclothes must be provided, and practically a new outfit. This again costs money, yet it is imperatively necessary that it should be done. For the sake of the reputation of the hospital and, much more, for the sake of the patients who enter it, no expense must be spared to ensure that thoroughness and efficiency are the guiding principles with the management. A rigid economy is exercised in every well-managed hospital; no penny is wasted; but at the same time the best is provided and care is taken that the patients who entrust themselves to the medical and nursing staffs get the utmost that science and art can do on their behalf.

### Increased Efficiency.

A third reason for the increased cost is that the standard of living in hospitals has risen. A hospital ward is no longer a pauper shelter; it is a place that can, and very often does, transcend the best private nursing home. There is no extravagance in this; it is merely the outcome of the carefully considered scheme for the benefiting of the largest number of patients in the shortest possible time. Where a patient's cure may be hastened by the use of some special though expensive remedy, or by the employment of some reliable but costly method of treatment, such remedy and such treatment must be provided. A modern hospital cannot be content with makeshifts; it must have the real article, of the best quality and workmanship. Only by adhering to this rule can it hope to continue to warrant the claim that it is a modern hospital.

### The Help given by Hospital Sunday.

The collections made on Hospital Sunday are of immense practical assistance to our voluntary hospitals. They are apportioned, finally, among the various institutions according to carefully considered principles, and in such distribution the work of the participating hospitals, the income of these institutions, the number of patients treated, and the general efficiency of the hospitals themselves are taken into account. No one who gives his contribution on Hospital Sunday need be under any apprehensions as to the spending of his share. It will not be frittered away; it will enable some institution to deal with a case, or a fraction of a case, in a thorough and effective manner. It will not go towards the furnishing of laboratories and the payment of school expenses; every penny of it will benefit the sick inmates of the institutions directly.

### The Bogey of Vivisection.

It is necessary to lay stress on these points. Even to-day, when Hospital Sunday is so well known and its principles have been so often explained, there are some people who do not scruple to attempt to dissuade intending contributors from giving their share to the funds collected on this day by holding up the scarecrow of vivisection. It is argued by these dissuaders that a hospital which has attached to it a medical school at which, presumably (for vivisection is not carried on in every medical school) vivisection is carried out, is unworthy of support because it allows such a school to remain attached to it. With the ethics of vivisection we have, at the moment, nothing whatever to do; every man or woman must individually decide whether the carrying out of experiments on living animals, for the possible benefit of mankind, is an immorality or not; it is purely a moral question. But the claims of the hospitals and the sick poor are incontestable; they claim support and help from everyone who is able to give such help. The inmates of a hospital are not responsible for what is done by the medical school attached to the institution, and it is unfair to make them suffer because the authorities of the hospital have decided that in their opinion vivisection is not an immorality but a necessary concomitant of research. This much is certain, namely, that no portion of the contributions on Hospital Sunday goes towards the upkeep of a medical school, and no person need therefore be deterred from contributing by the bogey which the anti-vivisectionists have raised.

